# Respiratory Motion Correction for Compressively Sampled Free Breathing Cardiac MRI Using Smooth *l*_1_-Norm Approximation

**DOI:** 10.1155/2018/7803067

**Published:** 2018-01-23

**Authors:** Muhammad Bilal, Jawad Ali Shah, Ijaz M. Qureshi, Kushsairy Kadir

**Affiliations:** ^1^Electrical Engineering Department, International Islamic University, Islamabad, Islamabad, Pakistan; ^2^Electrical Engineering Section, UniKL BMI, Gombak, Selangor, Malaysia; ^3^Electrical Engineering Department, Air University Islamabad, Islamabad, Pakistan

## Abstract

Transformed domain sparsity of Magnetic Resonance Imaging (MRI) has recently been used to reduce the acquisition time in conjunction with compressed sensing (CS) theory. Respiratory motion during MR scan results in strong blurring and ghosting artifacts in recovered MR images. To improve the quality of the recovered images, motion needs to be estimated and corrected. In this article, a two-step approach is proposed for the recovery of cardiac MR images in the presence of free breathing motion. In the first step, compressively sampled MR images are recovered by solving an optimization problem using gradient descent algorithm. The *L*_1_-norm based regularizer, used in optimization problem, is approximated by a hyperbolic tangent function. In the second step, a block matching algorithm, known as Adaptive Rood Pattern Search (ARPS), is exploited to estimate and correct respiratory motion among the recovered images. The framework is tested for free breathing simulated and* in vivo* 2D cardiac cine MRI data. Simulation results show improved structural similarity index (SSIM), peak signal-to-noise ratio (PSNR), and mean square error (MSE) with different acceleration factors for the proposed method. Experimental results also provide a comparison between* k-t* FOCUSS with MEMC and the proposed method.

## 1. Introduction

Compressed sensing (CS) has been successfully implemented to reduce the scan time of MR images [1–3]. Application of CS to transformed domain MR images includes brain [1], cardiac [4], and pediatric MR imaging [5]. According to CS approach, a sparse or compressible image can be recovered, by solving *l*_1_-*l*_2_ norm mixed optimization problem, from randomly undersampled data using a nonlinear recovery technique [2, 6]. Since the *l*_1_-norm is not differentiable everywhere, an approximation to the *l*_1_-norm is used in the reconstruction algorithm. Different approaches [1, 7] have been used to approximate *l*_1_-norm using some differentiable functions. Reference [7] uses hyperbolic tangent based function as an approximation of *l*_1_-norm to solve the CS recovery problem for static MR images.

Different types of motion during the data acquisition process cause artifacts like ghosting and blurring in the recovered cardiac MR images. In the presence of respiratory motion, high quality MR images can be produced by combining* k*-space profiles of the same cardiac phases in ECG-gated MR acquisition [8]. Addition of the same cardiac phases at different respiratory motions or states produces inconsistencies in* k*-space which results in motion artifacts in the combined reconstructed images. Sparsity, a necessary condition for CS, is also violated by this* k*-space profile combination [9]. Hence, to avoid periodic breath holds during the acquisition process and to take advantage of sparse signal recovery from undersampled data using CS methods, motion artifacts may need to be corrected. The combined approach of CS and motion correction has been implemented in [8–11]. Otazo et al. proposed 1D translational respiratory motion correction. Usman et al. introduced a reconstruction scheme for dynamic cardiac MRI by incorporating general motion framework directly into CS reconstruction. Their method uses data binning and intensity based nonrigid registration algorithm for estimating respiratory motions. Reference [11] proposed a CS based motion correction in the free breathing environment with multiple constraints. This method uses Demon based registration to estimate the motion between reference and other respiratory states.

Interframe motion estimation and compensation for time varying features of images has been used in video compression standards [12, 13]. These standards are based on different block matching algorithms [14] for motion estimation and compensation. Similar to video compression, dynamic MR images can be predicted by exploiting temporal redundancies between the images. Asif et al. proposed an algorithm, MASTeR [15], for the breath held condition, based on interframe motion to recover different cardiac MR images. MASTeR uses motion adaptive transform that models temporal sparsity using interframe motion estimation.* k-t* FOCUSS [16] also uses interframe motion estimation and compensation with a fixed reference frame during the image recovery process for breath held cardiac cine MRI.

In this article, a novel framework is presented for the recovery of highly undersampled free breathing cardiac MR images. Similar cardiac phases at different respiratory states are grouped like the frames of a video sequence. The Adaptive Rood Pattern Search (ARPS) technique, based on the interframe motion, is used to estimate and correct respiratory states among the grouped images. A two-step approach is adopted for the reconstruction of dynamic MR images. In the first step, free breathing cardiac phases without motion estimation are recovered from undersampled* k*-space data. Next, interframe motion between the reconstructed cardiac phases is calculated using ARPS to improve the image estimates iteratively. An approximation of the *l*_1_-norm penalty is used in the gradient descent algorithm to recover dynamic MR images. The adjustable parameters of the *l*_1_-norm approximation provide an extra benefit, as it can be adjusted to reflect the changing statistics of dynamic MR images. After the application of the proposed method, the combinations of similar cardiac phases at different respiratory states are clear and accurate as compared to the combined cardiac phase without motion estimation and correction.

The rest of the paper is organized as follows.  Section 2 discusses the preliminaries for interframe motion estimation in dynamic MRI and CS.  Section 3 describes the methodology of the proposed algorithm.  Section 4 gives the details of algorithm, Section 5 presents the simulation parameters and results followed by Section 6 that discusses the merits and demerits of the scheme, and Section 7 concludes the work.

## 2. Free Breathing Imaging Model and CS

Free breathing downsampled* k*-spaced data corrupted by motion states *d* = 1,2, 3,…, *D* for cardiac phase *n* = 1,2, 3,…, *N* is mathematically given as

(1) $$\begin{array}{c} y_{n}=\overset{D}{\underset{d=1}{\sum }}A_{d,n}Fx_{d,n}=\overset{D}{\underset{d=1}{\sum }}\Phi x_{d,n}, \end{array}$$

where **x**_*d*,*n*_ is a two-dimensional complex MR image vector of length *T* representing a cardiac phase *n* at respiratory state *d*, **F** is a Fourier operator that transforms an image to* k*-space, **A**_*d*_ is a random variable-density undersampling mask, different for all respiratory states and Φ = **AF** is a sensing matrix, and **y**_*n*_ is a combined* k*-space measurement vector of length *C* for* n*th cardiac phase acquired for all respiratory positions. A single cardiac phase* n* at respiratory state* d* in a specific heart cycle can be given as

(2) $$\begin{array}{c} y_{d,n}=A_{d,n}{Fx}_{d,n}=\Phi x_{d,n}. \end{array}$$

The reduction or acceleration factor for MR images is given by *R* = *T*/*C*. By increasing *R*, the system in (1) becomes highly underdetermined. Compressed sensing solves such underdetermined system of equations effectively to recover MR images. The recovery of a sparse signal **x** ∈ *ℝ*^*T*^ using CS can be achieved by solving the following convex optimization problem:

(3) minx fx≔Φx−y22+λx1,

where *λ* ∈ *ℝ*^+^ is the Lagrangian that provides a balance between sparsity and data consistency. The *l*_2_-norm keeps the solution consistent with the data and the *l*_1_-norm given by ‖**x**‖_1_≔∑_*i*=1_^*T*^|*x*_*i*_| encourages sparsity in solution [2].

## 3. Methods

### 3.1. Smooth *l*_1_-Norm Approximation

CS algorithms recover sparse signals or images by solving the *l*_1_-norm regularized optimization problem such as that given in (3). In this article, we use gradient descent algorithm for solving the optimization problem with wavelet based penalty term. Nondifferentiability of the *l*_1_-norm at origin excludes the usage of mostly optimization approaches for the solution. Reference [1] uses an approximation of $\left|x\right|\approx \sqrt{xx^{*}+\mu }$ with *x*^*∗*^ the complex conjugate and *μ* a positive smoothing factor. Reference [7] proposes a new smooth function for approximating the *l*_1_-norm, used in this paper, which is given below

(4) $$\begin{array}{c} \left|\;x\;\right|\approx x\tanh\left(\;\gamma x\;\right). \end{array}$$

This function better approximates the absolute value and provides extra flexibility of adjusting the slope at the origin with the proper selection of *γ* and makes it more suitable for dynamic images. Mostly MR images are sparse in transformed domain so the modified version of cost function given in (3) for transformed MR images is

(5) minx fx≔Φx−y22+λΨx1,

where Ψ is a wavelet operator that transforms the image to a sparse domain.

In this article, we propose an iterative algorithm that uses the following approximation for the *l*_1_-norm penalty:

(6) $$\begin{array}{c} {\left\|\;x\;\right\|}_{1}\approx \overset{T}{\underset{i=1}{\sum }}x_{i}\tanh\left(\;\gamma x_{i}\;\right)=\overset{T}{\underset{i=1}{\sum }}\alpha \left(\;x_{i}\;\right), \end{array}$$

where *α*(*x*_*i*_) = *x*_*i*_tanh⁡(*γx*_*i*_). The update equation for the algorithm, derived using the steepest descent method for a sparse vector **x** ∈ *ℝ*^**T**^, is

(7) $$\begin{array}{c} x_{i+1}=x_{i}-\eta \nabla f\left(\;x_{i}\;\right), \end{array}$$

where *η is *positive valued step size and ∇ is the gradient operator that differentiates the cost function *f*(**x**) at *i*th iteration. During each iteration, shrinkage given in (8) is applied in the wavelet domain after (7) to reconstruct the MR images.

(8) $$\begin{array}{c} T_{\beta }\left(\;z\;\right)=\max\left\{\;\left|\;z\;\right|-\beta ,0\;\right\}\cdot \mathrm{sgn}\left(\;z\;\right), \end{array}$$

where *β* is a thresholding parameter. By incorporating the approximation ‖**x**‖_1_ ≈ ∑_*i*=1_^*T*^*α*(*x*_*i*_), the cost function can be written as

(9) $$\begin{array}{c} f\left(\;x\;\right)=\frac{1}{2}{\left\|\;\Phi x-y\;\right\|}_{2}^{2}+\lambda \overset{T}{\underset{i=1}{\sum }}\alpha \left(\;x_{i}\;\right). \end{array}$$

The gradient of the cost function is easy to compute:

(10) $$\begin{array}{c} \nabla f\left(\;x\;\right)=\Phi^{T}\left(\;\Phi x-y\;\right)+\lambda \overset{T}{\underset{i=1}{\sum }}\alpha^{\prime }\left(\;x_{i}\;\right) \end{array}$$

with

(11) $$\begin{array}{c} \alpha^{\prime }\left(\;x_{i}\;\right)=\tanh\left(\;\gamma x_{i}\;\right)+x_{i}\gamma \left(\;1-\tanh^{2}\left(\;\gamma x_{i}\;\right)\;\right). \end{array}$$

### 3.2. Respiratory Motion Based Dynamical System

Two main problems with free breathing cardiac MRI are as follows.

(1) Blurring artifacts are generated by the combination of* k*-space samples for the same cardiac phases at different respiratory states.

(2) The combination of* k*-space data in free breathing decreases the sparsity level.

In this article, we use interframe motion to estimate the respiratory states between the same cardiac phases. Video standards MPEG and H.264 [12, 13] have successfully exploited interframe motion for compression. In the dynamic MRI images, pixels are not significantly displaced in the neighboring frames. Pixel locations can be predicted using interframe motion estimation. Temporal redundancy among the frames is advantageous for the prediction of pixel locations. Let **x**_*d*,*n*_ and **x**_*d*,*n*+1_ be images having *n*th cardiac phases at respiratory states* d* and *d* + 1, respectively. The pixel values of **x**_*d*_ at location (*a*, *b*) are closest to the pixel values at (*a* + Δ*a*, *b* + Δ*b*) in **x**_*d*+1_. The displacement of all pixels in **x**_*d*,*n*_ from (*a*, *b*) to (*a* + Δ*a*, *b* + Δ*b*) in **x**_*d*+1,*n*_ is represented by motion vectors (Δ*a*, Δ*b*). According to [2], cardiac phase **x**_*d*,*n*_ at* d*th respiratory state can be generated from the cardiac phase **x**_*d*+1,*n*_ at (*d* + 1)th respiratory state by the following equation:

(12) $$\begin{array}{c} x_{d,n}=M_{d+1,n}x_{d+1,n}+m_{d,n}, \end{array}$$

where **M**_*d*+1,*n*_ is a backward transformation that uses information about the physical changes between two datasets of the same cardiac phases. The motion compensated residual is computed by taking the difference between predicted and compensated image. Using the transformation **M**_*d*+1,*n*_, a motion dependent linear system can be written by combining (1) and (12) as follows:

(13a) yn=∑d=1DAd,nFxd,n,

(13b) md,n=Md+1,nxd+1,n−xd,n.

**Figure 1:**
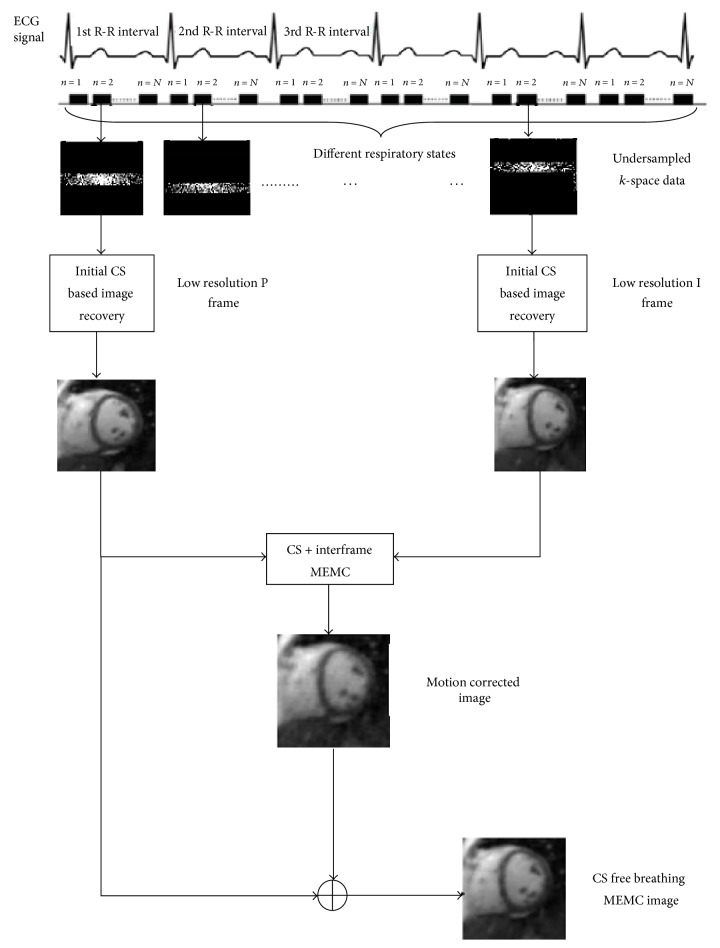
A presentation of initial CS recovery and CS-MEMC recovery steps of the proposed method. For simplicity, only two heart phases with different respiratory states are shown with six heart beats and *N* cardiac phases. Low resolution P and I frames are generated primarily by CS. Using ARPS block matching algorithm, motion is estimated and corrected to produce a motion corrected image. The CS-free breathing MEMC image is generated by combining a low resolution P frame and motion corrected image.

To recover the cardiac phases **x**_*d*,*n*_, we solve (13a) and (13b) by exploiting sparse structure in **m**_*d*,*n*_ and **x**_*d*,*n*_. The process of complete high resolution image generation is shown in Figure 1. The data is acquired in segmented fashion because MRI is a slow imaging modality. During the data scanning process, a limited* k*-space sample is recorded at each heart phase in all cardiac cycles. To simulate this condition, each cardiac phase at different respiratory states is multiplied with different sampling matrix **A**_*d*,*n*_.

### 3.3. Initial CS Reconstruction

A two-step approach is adopted to recover motion free cardiac phases. In the 1st step, images with motion effects are reconstructed from undersampled* k*-space data independently. For the recovery of dynamic cardiac images, the iterative algorithm, mentioned as Algorithm 1, optimizes the cost function given in (9) with the approximation given in (4). Wavelet based soft thresholding is used for the recovery of the* N*th cardiac phase for each respiratory state. Daubechies-4 (db4) wavelet is used to exploit the transformed domain sparsity.

### 3.4. Interframe Motion Estimation and Correction (MEMC)

In the 2nd step, interframe motion estimation and correction is performed from a pair of CS recovered images and divided into two substeps.

*(a) Motion Estimation*. Exploit initially CS reconstructed images to estimate or refine interframe motion and the motion transformation **M** as follows. Cardiac phase in the 1st R-R ECG interval is taken as a P frame (frame to be predicted) and cardiac phases in subsequent R-R intervals are taken as an I frame (reference frame). The P and I frame are borrowed terminology from video compression. To estimate the motion between the 2nd cardiac phases of the 1st and 4th R-R interval, for example, we take the 2nd cardiac phase of the 1st interval as a P frame and the 2nd cardiac phase at different respiratory state in the 4th R-R interval as an I frame.

*(b) Motion Correction*. After finding motion vectors using ARPS block matching algorithm, we generate the corrected image from I frame and with the help of motion vectors. For the refinement of motion corrected image, solve the following optimization problem written for (13a) and (13b):

(14a) minx fxd,n≔Φxd,n−yd,n22+λmd,n1,

where **m**_*d*,*n*_ is given in (13b) and its *l*_1_-norm approximation is

(14b) $$\begin{array}{c} {\left\|\;m_{d,n}\;\right\|}_{1}=\overset{E}{\underset{e=1}{\sum }}{\left(\;m_{d,n}\;\right)}_{e}\tanh\left(\;\gamma {\left(\;m_{d,n}\;\right)}_{e}\;\right). \end{array}$$

At the final step, we generate the image of 2nd cardiac phase by combining P frame and motion corrected image to get an image with high temporal and spatial resolution.

## 4. Proposed Algorithm

Steps involved in the reconstruction of MR motion corrected images are given in Algorithm 1.

## 5. Experimental Setup and Results

The proposed method was tested on simulated data generated by the MRXCAT framework [17] and on fully sampled, free breathing, cine MRI data. The recovered images for CS-free breathing motion corrected were compared with CS-free breathing images and with CS-breath held images. All CS images were recovered in MATLAB (R2012a, MathWorks Inc., Natick, MA) using the proposed hyperbolic tangent based surrogate function to solve the nondifferentiability problem of the *l*_1_-norm penalty. In the gradient descent algorithm, step size *η* in an update equation was chosen empirically. Parameter values used in the algorithm are *β* = 0.005, *η* = 0.9, and *γ* ≥ 10. The same values for *β*, *η*, and *γ* were used for initial CS reconstruction and for the final interframe motion estimation and compensation.

The structural similarity index (SSIM) [18], peak signal-to-noise ratio (PSNR), and mean square error (MSE) were used for quantitative comparison between CS-free breathing reconstruction with motion correction and that without motion correction. The PSNR and MSE of complete image and ROI for CS-free breathing motion corrected and CS-free breathing were calculated as

(15) PSNR in dB=10 log10⁡MAXc2MSE,MSE=1z×z∑i=0Z−1 ∑j=0Z−1Cij−Rij2,

where MAX_*c*_ is a maximum pixel value of the current image having dimensions of *Z* × *Z* and *C*_*ij*_ and *R*_*ij*_ are pixels being compared with current and reference cardiac phases, respectively. The ARPS block matching algorithm was used for interframe respiratory motion estimation between the reference image and the current image. Diastolic, middle of systolic and diastolic, and systolic heart phases at different respiratory states were used for both simulated images and clinical data.

The MRXCAT, a Matlab software for numerical simulation of cardiac MRI, is used for generating free breathing and breath held images. For the MRXCAT, the following parameters were used: reconstruction matrix size of 256 × 256, 24 cardiac phases in the presence of respiratory motion, with an image resolution of 1 × 1 × 1 mm^3^, TE = 1.5 ms, TR = 3 ms, and flip angle = 60°. In real free breathing cardiac cine MRI, fully sampled ECG-gated data was acquired on a Philips 1.5 T scanner (b-SSFP). Reconstruction matrix size of 256 × 256, 6 cardiac cycles with 24 cardiac phases in each cycle, and an image resolution of 2.5 × 2.5 × 8 mm^3^ were used.

For comparison of the proposed method with* k-t* FOCUSS, we used the following data: a short-axis MRI scan (images shown in Figure 5) was acquired using a GE 1.5 T Twin Speed scanner (R12M4) with a 5-element cardiac coil and a FIESTA/FastCARD cine SSFP sequence. Scan parameters were selected as follows: TE: 2.0 ms, TR: 4.1 ms, flip angle: 45°, FOV: 350 × 350 mm, slice thickness: 12 mm, 8 views per segment, 224 phase-encoding lines, 256 read-out samples, and 16 temporal frames. To emulate the estimation of sensitivity maps from a prescan, we acquired a separate scan (which we assume to be a prescan) with identical scan parameters and estimated sensitivity maps as follows: Half of the (high frequency)* k*-space samples from each coil were removed from the prescan via a smoothing filter followed by an inverse Fourier transform to obtain smoothed images for each coil. To estimate the sensitivity maps, we divided each smoothed coil image by the root sum of squares of all coil images.

**Figure 2:**
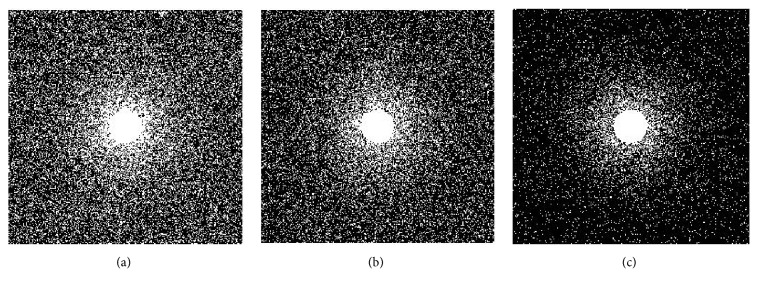
Variable-density sampling patterns for different acceleration rates (*R*): (a) *R* = 3, (b) *R* = 4, and (c) *R* = 8.

The acquired data were retrospectively undersampled for acceleration rates *R* = 2 (50% of samples), 3 (33% of samples), and 8 (12.5% of samples) using variable-density random undersampling method. Sampling masks for different acceleration rates are shown in Figure 2. The sampling mask randomly selects more samples from the low frequencies of the* k*-space data and fewer samples from the high frequencies of the* k*-space data.

**Figure 3:**
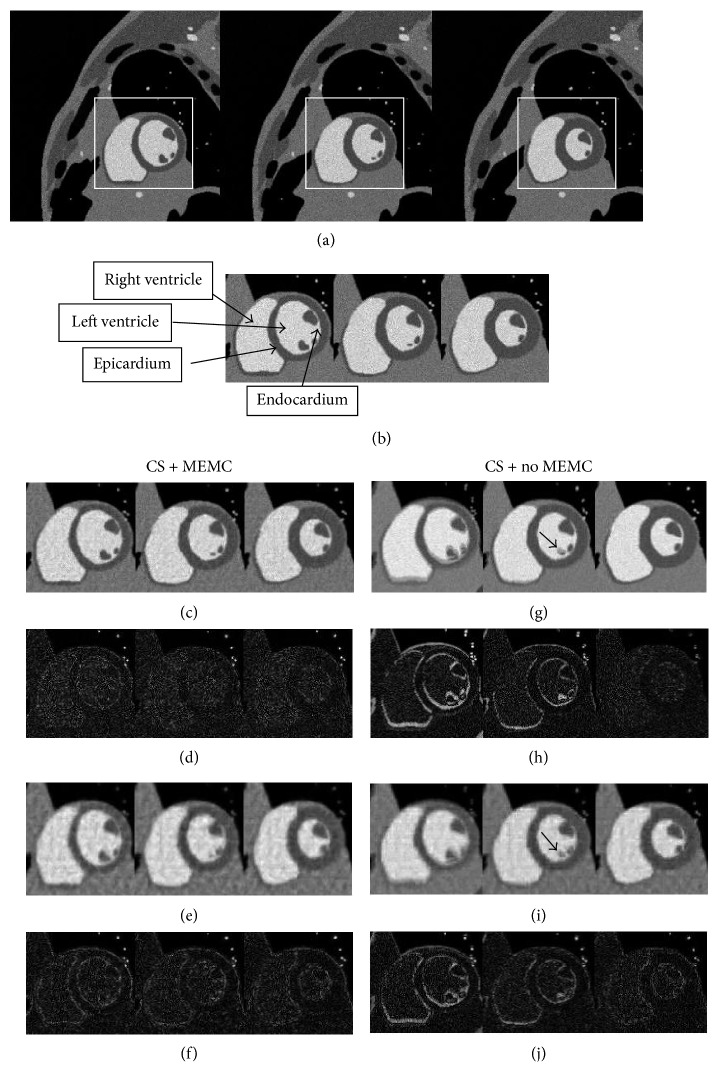
Comparison of recovered images with and without respiratory motion estimation for simulated data: frames 1, 5, and 12 (left to right). (a) Gold standard images from full* k*-space breath held data. (b) Spatial region of interest (ROI). (c) Reconstruction using the proposed technique (CS + MEMC) at *R* = 2. (d) Difference between estimated images (c) and (b). (e) Reconstruction using the proposed technique (CS + MEMC) at *R* = 8. (f) Difference between estimated images (e) and (b). (g) Reconstruction with CS + no MEMC at *R* = 2. (h) Difference between estimated images (g) and (b). (i) Reconstruction CS + no MEMC at *R* = 8. (j) Difference between estimated images (i) and (b).

Figure 3 provides a comparison of the CS-free breathing motion corrected (CS + MEMC), CS-free breathing (CS + no MEMC), and breath held for the short-axis MRI images generated from the MRXCAT simulation software at the reduction factors 2 and 8. Figure 3(a) illustrates frames 1, 5, and 12 out of 24 frames in a sequence, produced from fully sampled breath held* k*-spaced data. Most of the changes occur in the heart region, enclosed in the white box in (a), and are taken as a region of interest (ROI). Figure 3(b) shows the ROI, specifying left and right ventricles with endocardium and epicardium. Figures 3(c) and 3(e) show the proposed method recovery (CS + MEMC) at the reduction factors 2 and 8, respectively. Figures 3(d) and 3(f) represent the difference between breath held and estimated images for the proposed method. Figures 3(g) and 3(i) show CS + no MEMC at the reduction factors 2 and 8, respectively. Figures 3(h) and 3(j) represent the difference between breath held and estimated images with CS + no MEMC.

Images recovered by the proposed method show significant improvement as compared to the image recovery without MEMC at both reduction factors. Motion artifacts like ghosting and blurring can be seen in Figures 3(g) and 3(i) pointed by the black arrows. The proposed method eliminated ghosting and blurring effects and achieved high spatial and temporal accuracy as shown in Figures 3(c) and 3(e). The elimination of motion artifacts provides sharp endocardium and epicardium borders. This sharpness is very important in the clinical interpretation of ventricular dynamics. The improved recovery of the proposed method is also evident from difference images.

**Figure 4:**
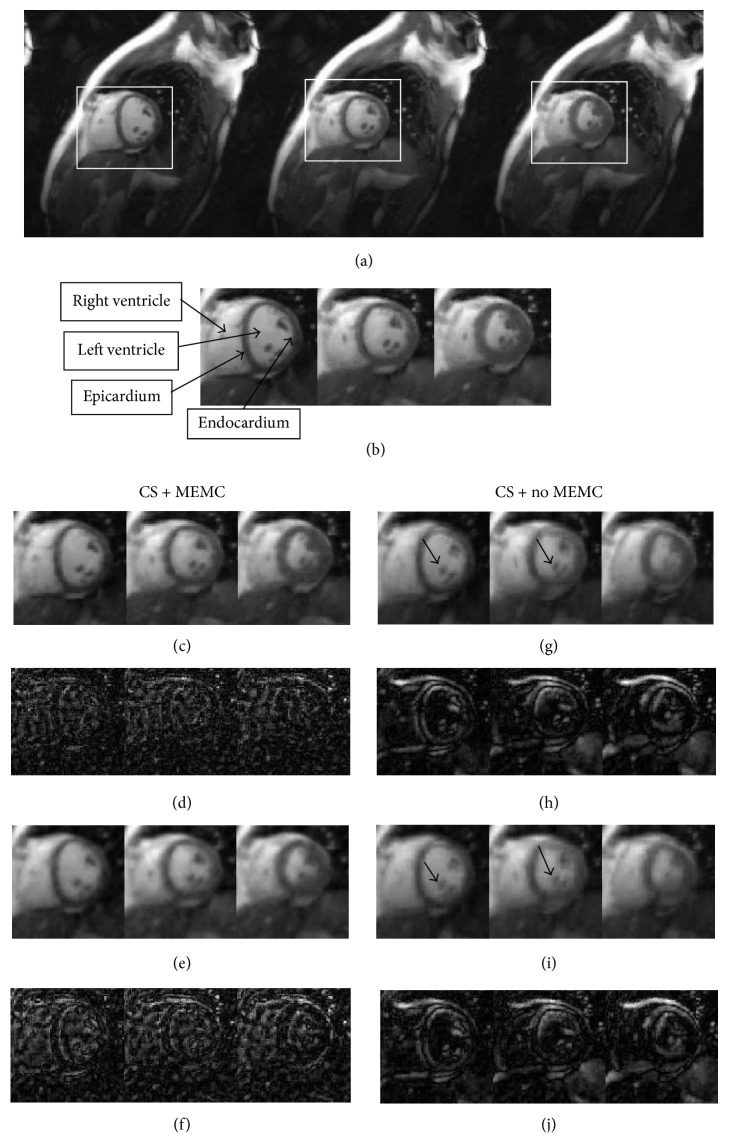
Comparison of recovered images with and without respiratory motion estimation for clinical data: frames diastolic, middle of diastolic and systolic, and systolic (left to right). (a) Gold standard images from full* k*-space data. (b) ROI. (c) Reconstruction using the proposed technique (CS + MEMC) at *R* = 3. (d) Difference between estimated images (c) and (b). (e) Reconstruction using the proposed technique (CS + MEMC) at *R* = 8. (f) Difference between estimated images (e) and (b). (g) Reconstruction with CS + no MEMC at *R* = 3. (h) Difference between estimated images (g) and (b). (i) Reconstruction CS + no MEMC at *R* = 8. (j) Difference between estimated images (i) and (b).

Figure 4 presents the comparison of the proposed method and CS + no MEMC for the short-axis MRI images at the reduction factors 3 and 8.

Figure 4(a) illustrates complete dataset of a diastolic, middle of diastolic and systolic, and systolic frames in a sequence, produced from clinically observed fully sampled* k*-spaced data. Figure 4(b) shows the ROI, enclosed in rectangular box in (a), representing left and right ventricle and epicardium and endocardium. Figures 4(c) and 4(e) show the proposed method recovery at reduction factors 3 and 8, respectively. The results of CS-free breathing without MEMC at reduction factors 3 and 8 are illustrated in Figures 4(g) and 4(i), respectively. For clinical data, images generated by the proposed method show significant improvement as compared to the image recovery without MEMC at both reduction factors. Motion artifacts like ghosting and blurring can be seen in Figures 4(g) and 4(i) pointed by black arrows. The systolic phase recovered by proposed method in (c) and (e) is very close and clear to fully sampled ROI in (b) as compared to (g) and (i) where not only are the images ghosted and blurred, but also the heart walls are displaced from their true location. Sharpness of epicardium and endocardium is also prominently visible in images recovered through the proposed method.

**Figure 5:**
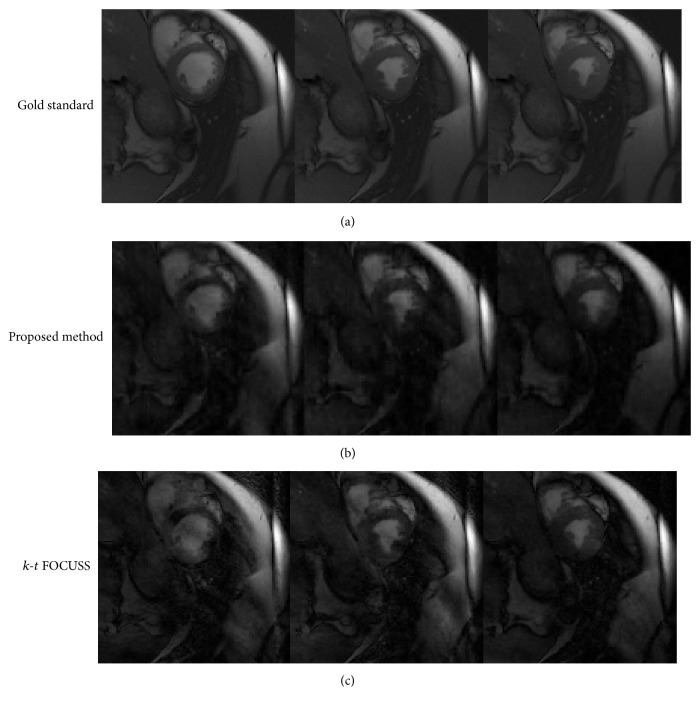
Comparison of recovered images in pixel domain for proposed method and* k-t* FOCUSS with MEMC data at a reduction factor of 4. (a) Fully sampled breath held* k*-space data. (b) Recovered images with proposed method. (c) Reconstructed images for* k-t* FOCUSS with MEMC.

Figure 5 illustrates the comparison of the proposed method (CS + MEMC) and* k-t* FOCUSS with MEMC for the short-axis MRI dataset at reduction factor 4.  Figure 5(a) shows frames 1, 13, and 10 (from left to right) out of the 16 frames in the sequence, calculated from the fully sampled breath held* k*-space data. Using* k*-space tutorial [19], motion-corrupted images are generated from the short-axis MRI dataset.  Figure 5(b) presents the proposed technique reconstructions at reduction factor 4. The results for* k-t* FOCUSS with MEMC at reduction factor 4 are presented in Figure 5(c). The proposed method reconstruction shows significant improvement and less random noise than* k-t* FOCUSS with MEMC reconstruction. Furthermore,* k-t* FOCUSS with MEMC reconstructions contains motion artifacts (visible with bright regions), while the proposed method reconstructions are much cleaner. Tables 1, 2, and 3 provide a comparison of the proposed method and* k-t* FOCUSS for performance metrics such as SSIM, PSNR, and MSE. The numerical values of metrics for selected frames 1, 10, and 13 show that the proposed method outperforms* k-t* FOCUSS with MEMC.

**Figure 6:**
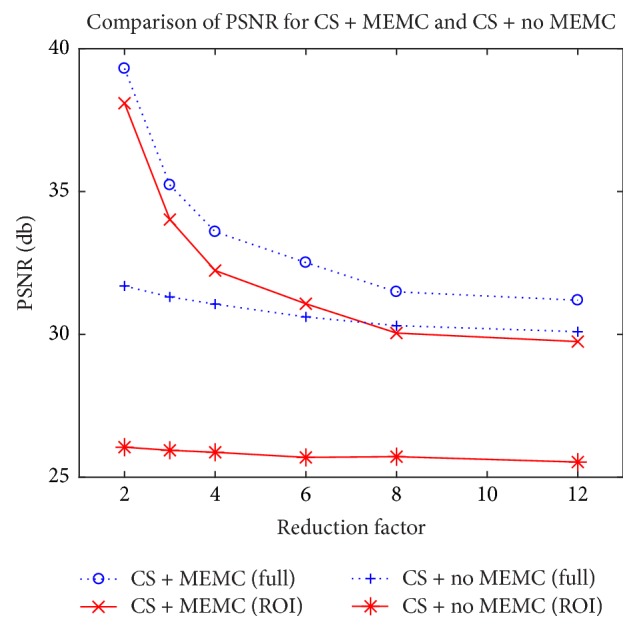
Performance comparison of PSNR at different reduction factors for CS-free breathing (CS + no MEMC) and CS-free breathing motion corrected (CS + MEMC). Dotted lines depict PSNR over the full image and solid lines show PSNR in the region of interest (ROI).

A plot for PSNR at different reduction factors for CS-free breathing and CS-free breathing motion corrected is shown in Figure 6. It is drawn for recovered images shown in Figure 4. Dotted lines denote PSNR over the ROI and the solid line shows it over the entire image. The curves show that CS-free breathing motion corrected (the proposed method) is far better than the CS-free breathing at all reduction factors for both the full reconstruction and the reconstructions of ROI. Even at higher reduction factor like 12, PSNR for ROI is 4 db better in CS-free breathing with MEMC as compared to CS-free breathing without MEMC.

**Figure 7:**
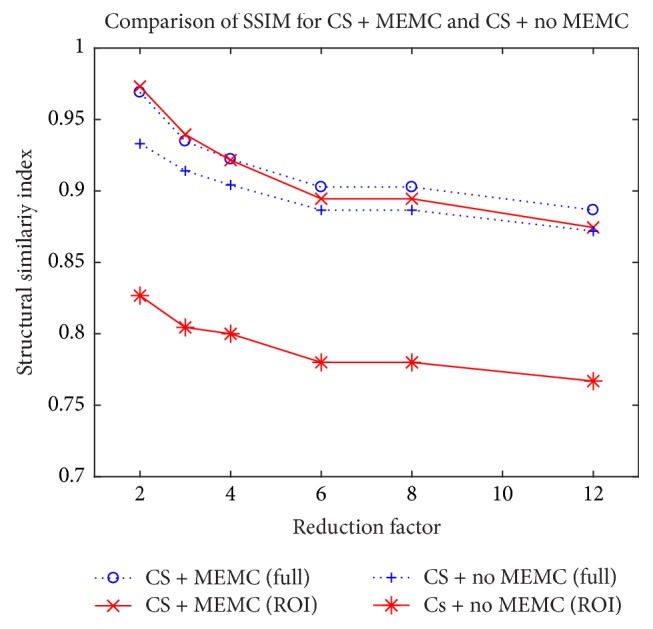
Performance comparison of SSIM at different reduction factors for CS-free breathing without MEMC and CS-free breathing with MEMC. Dotted lines depict SSIM over the full image and solid lines show SSIM in the region of interest (ROI).

To show how the recovered images, with and without MEMC, are similar to the fully sampled images, we used SSIM. A plot for SSIM at different reduction factors for CS-free breathing and CS-free breathing motion corrected is shown in Figure 7. The plot is drawn for clinical data of Figure 4. Solid lines denote SSIM over the ROI and dotted line shows it over the entire image (full image). The curves illustrate that the images recovered with the proposed method are more similar to a gold standard as compared to CS-free breathing without MEMC at all reduction factors.

**Figure 8:**
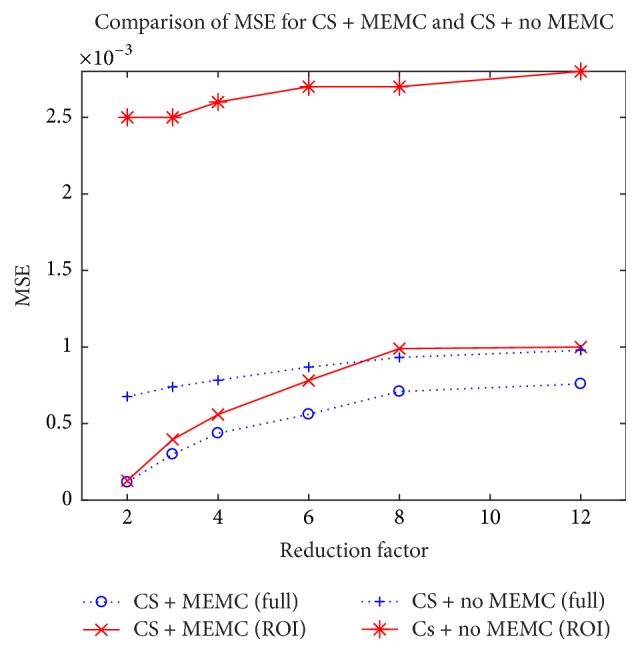
Performance comparison of MSE at different reduction factors for CS-free breathing and CS-free breathing motion corrected. Dotted lines depict MSE over the entire region and solid lines depict MSE in the region of interest (ROI).

A plot for reconstruction mean square error (MSE) at different reduction factors for CS-free breathing and CS-free breathing motion corrected is illustrated in Figure 8. The plot is drawn for clinical data of Figure 4. Solid lines denote MSE over the ROI and dotted line shows it over the entire image. The curves illustrate that images recovered with proposed method have smaller MSE in comparison with CS-free breathing without MEMC at all reduction factors for both entire region and ROI.

## 6. Discussion

Interframe motion estimation and compensation has been used to improve cardiac images in breath held condition and at the same respiratory states. The proposed CS-free breathing motion corrected framework exploits temporal redundancy among the same cardiac phases at different respiratory states (free breathing). The source of improvement is the use of a novel smooth *l*_1_-norm approximation and inclusion of sparse residual in the basic cost function of (3).

This article suggests a CS-free breathing motion corrected recovery from cardiac cine MR data acquired below the Nyquist sampling rate. The proposed framework is a combination of interframe motion estimation technique and compressed sensing. The ARPS block matching algorithm is used for respiratory motion estimation and compensation. A simple gradient descent algorithm, with the *l*_1_-norm approximated by hyperbolic tangent function, is used to reconstruct the motion corrected images. The proposed method is implemented for 2D ECG-gated cardiac cine MRI for both simulation and clinical data. Implementation parameters are discussed in an experimental setup section.

The proposed method requires higher computations due to an iterative nature of the algorithm. To estimate motion corrected image, the algorithm needs to compute motion operators **M** and **x** alternatively multiple times. The ARPS introduces interpolation error during the prediction process of motion corrected image. There is a need to investigate motion estimation schemes with reduced interpolation error during the process of motion estimation and correction. This error is inherent in all estimation schemes and is not a topic in this article.

In the presented scheme, motion corrected images are produced from a fixed reference frame. In the future, motion estimation can be done from adjacent frames in both forward and backward direction and other CS recovery approaches can be used. Further research might be required to find its usage in 3D dynamic cardiac MRI. The proposed method will require modification for arrhythmic patients because heart rate variability is not considered in this work. Similar cardiac phases at different respiratory states are chosen visually from a sequence of cardiac MRI frames. Data binning might be used for selection of cardiac phases at different respiratory states.

## 7. Conclusion

In this article, we proposed a method for respiratory motion correction in ECG-gated free breathing cardiac MRI. Interframe motion estimation was used to estimate the respiratory motion between the same cardiac phases, but at different respiratory states. The block matching algorithm was used for MEMC. A gradient descent algorithm based on flexible *l*_1_-norm approximation was used for the recovery of MR images free from motion artifacts and close to the true MR images. Standard metrics like SSIM, PSNR, and MSE at different reduction factors were observed to be superior for the proposed method as compared to the results obtained without MEMC and* k-t* FOCUSS.

## Figures and Tables

**Algorithm 1:**
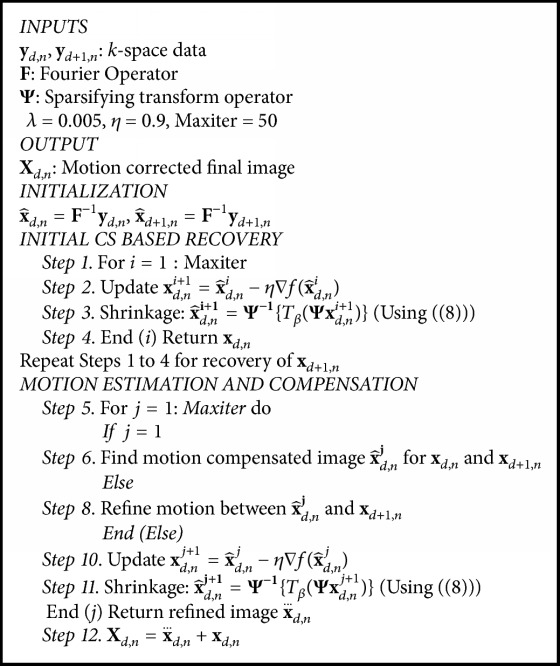
The proposed algorithm.

**Table 1:** SSIM comparison for the proposed method and *k-t* FOCUSS with MEMC.

Technique	Diastolic (frame #1)	Systolic (frame #10)	Middle (frame #13)
The proposed method	0.7319	0.8687	0.8260
*k-t* FOCUSS	0.7004	0.8368	0.7302

**Table 2:** PSNR (db) comparison for the proposed method and *k-t* FOCUSS with MEMC.

Technique	Diastolic (frame #1)	Systolic (frame #10)	Middle (frame #13)
The proposed method	29.9443	34.2610	31.1526
*k-t* FOCUSS	25.4316	32.3289	26.2374

**Table 3:** MSE comparison for the proposed method and *k-t* FOCUSS with MEMC.

Technique	Diastolic (frame #1)	Systolic (frame #10)	Middle (frame #13)
The proposed method	0.001	3.7488*e* − 4	7.6690*e* − 4
*k-t* FOCUSS	0.002	5.8494*e* − 4	0.0024
